# Piezoelectric Microstructured Fibers *via* Drawing of Multimaterial Preforms

**DOI:** 10.1038/s41598-017-01738-9

**Published:** 2017-06-06

**Authors:** Xin Lu, Hang Qu, Maksim Skorobogatiy

**Affiliations:** École Polytechnique de Montréal, Montreal, Québec H3T 1J4 Canada

## Abstract

We demonstrate planar laminated piezoelectric generators and piezoelectric microstructured fibers based on BaTiO_3_-polyvinylidene and carbon-loaded-polyethylene materials combinations. The laminated piezoelectric generators were assembled by sandwiching the electrospun BaTiO_3_-polyvinylidene mat between two carbon-loaded-polyethylene films. The piezoelectric microstructured fiber was fabricated *via* drawing of the multilayer fiber preform, and features a swissroll geometry that have ~10 alternating piezoelectric and conductive layers. Both piezoelectric generators have excellent mechanical durability, and could retain their piezoelectric performance after 3 day’s cyclic bend-release tests. Compared to the laminated generators, the piezoelectric fibers are advantageous as they could be directly woven into large-area commercial fabrics. Potential applications of the proposed piezoelectric fibers include micro-power-generation and remote sensing in wearable, automotive and aerospace industries.

## Introduction

Driven by the ever-growing market of the personal wearable products such as on-garment displays, health-monitoring sensors^1^, virtual-reality devices, smart watches and bracelets, and intelligent glasses, extensive effort has been devoted to the R&D of soft and wearable electronics^2^. The development of wearable and portable electronics has inspired much work^3^ in the design and fabrication of flexible fibers which could be used as power sources or sensor components. Among all of these fiber generators or sensors, piezoelectric fibers that operate based on piezoelectric effect^4, 5^ are especially attractive, because they could convert mechanical vibrations accessible in our daily life (i.e. walking^6^, air flow^7^ and heart beating^3, 8^) into electrical signals. Another application of piezoelectric generators is related to automotive or aerospace industries^9, 10^. Piezoelectric generators or sensors are implanted on the airplanes and vehicles, for the purpose of structural integrity monitoring^11^, as well as powering the on-board electronic systems such as wireless sensor networks (WSNs) with low-power consumption^12, 13^.

To date, a number of piezoelectric fibers have been demonstrated. A straightforward route for fabrication of the piezoelectric fibers is to directly grow or wet-extrude piezoelectric ceramic materials such as ZnO nanorods or nanowires (NWs), BaTiO_3_ (BTO) nanostructures and Pb(Zr_0.52_Ti_0.48_)O_3_ (PZT) NWs along a metallic wire/microfiber^14–16^. For instance, Yin *et al*. report a piezoelectric fiber by depositing ZnO nanorods on a copper wire^17^. Qiu *et al*. demonstrate a Pb(Nb,Ni)O_3_-Pb(Zr,Ti)O_3_ (PNN-PZT) piezoelectric ceramic fiber by extruding a mixture of PNN-PZT powder and organic solvent together with a 50 μm Pt core^18^. However, for these fibers, frequent and intensive mechanical movements may potentially damage the fiber structure (e.g. the continuous bending can make the piezoelectric layers cracking, and even peeling off from the fiber core). Thus, the as-fabricated fiber generators typically suffer from poor mechanical reliability, which makes them unsuitable for truly wearable applications. To improve robustness of the fibers, Zhang *et al*. have coated a thin layer of polydimenthylsiloxane (PDMS) on the roots of ZnO NWs using surface-coating combined with plasma-etching^16^. An alternative route for fabrication of the piezoelectric fibers involves the utilization of piezoelectric polymers. In principle, piezoelectric fibers based on piezoelectric polymers usually feature better mechanical robustness and flexibility^19^. Among all of these piezoelectric polymers, poly(vinylidene fluoride) (PVDF) and poly(vinylidene fluoride-co-trifluoroethylene) (PVDF-TrFE) are particularly attractive due to their ease of production, high chemical resistance, superior flexibility, and good piezoelectric performance^4^. Most of the existing piezoelectric polymer fibers are fabricated by melt-spinning or extrusion. For example, Lund *et al*. reported the melt-spinning of a PVDF-yarn with a conductive carbon black/polypropylene (CB/PP) core^20^. Using a similar method, Bian *et al*. demonstrated a metal-core piezoelectric fiber that had a PVDF sheath and a molybdenum filament core^21^. Recently, Martins *et al*. used the melt-coextrusion method to fabricate a piezoelectric fiber that has a piezoelectric PVDF layer sandwiched by two polypropylene-based conductive polymers^22^. Note that the piezoelectric fibers fabricated by traditional spinning methods typically adopt a simple core-sheath structure, in which a conductive filament constitutes the core, and a piezoelectric polymer layer constitutes the sheath. Besides, an additional conductive layer should be coated on the fiber as an external electrode that may limit the lifetime and applications of the as-spun fibers, since the metallic layer under repeated mechanical deformations generally results in fracture. In the case of fiber spinning/stretching, the stress applied on the fiber can induce the conversion of the nonpolar *α* into the ferroelectric *β* phase. Additionally, an electrical poling of the piezoelectric fibers during (or after) the spinning process can considerably promote the phase transformation.

**Figure 1:**
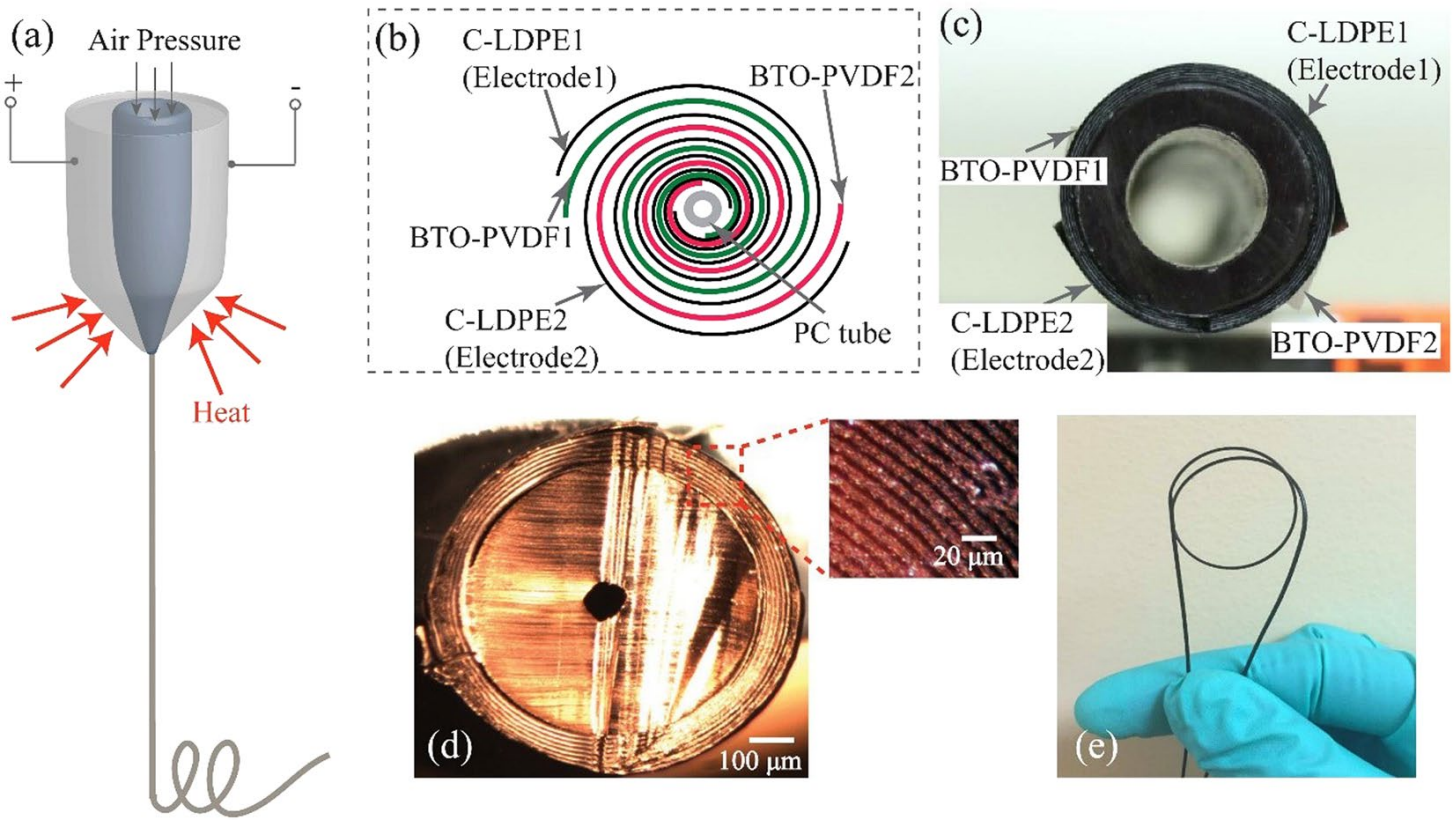
Fabrication of the piezoelectric microstructured fibers *via* drawing of the multimaterial preforms. (**a**) Schematic of the fabrication process of a piezoelectric fiber. (**b**) Schematic of the multilayer structure in the fiber preform and in the microstructured fiber. (**c**) Photo of a preform cross section. (**d**) Photo of a cross section of the piezoelectric microstructured fiber. Insert: the magnified view of a multilayer structure. (**e**) Photo of a piezoelectric microstructured fiber.

Fabrication of the piezoelectric polymer fibers could be also achieved by fiber drawing technique (Fig. 1a). In this method, kilometer-long piezoelectric fibers of sub-millimeter diameters are thermally drawn from a geometrically complex multimaterial fiber preform. The fiber perform could be assembled using a variety of materials such as thermoplastic polymers, glass and even metals. The preform is then heated in a vertical furnace and drawn into the extended lengths of fiber. The resultant fiber generally preserve the perform structure but with a much smaller cross-section dimension. By engineering the fiber inner microstructure and optimizing the drawing conditions, the fibers produced from fiber drawing technique would provide various electrical functionalities as well as great mechanical flexibility. Egusa *et al*.^23^ reported the fabrication of piezoelectric fibers based on PVDF-TrFE using the fiber drawing technique. In that fiber, the PVDF-TrFE layer was sandwiched between two carbon-loaded polycarbonate layers and assembled with Tin microfilaments as the electrodes. The fiber also covered with polycarbonate shell for the protective cladding. The limitations of these piezoelectric fibers are evident, as they use very expensive PVDF-TrFE material, and the integration of Tin microfilaments reduces the fiber reliability. Kanik *et al*. fabricated piezoelectric PVDF micro- and nano- ribbons using iterative size reduction technique based on thermal fiber drawing^24^. In order to obtain spontaneously polar *γ* phase PVDF, one should redraw the same fiber multiply. At this point, the robustness and reproducibility of this fabrication technique would be major concerns; however, there were no further evaluations and reports of this fabrication method. Also, consecutive re-drawings of the same fiber would be time and labor consuming. Instead, a PVDF can be impregnated with piezoelectric ceramics such as BTO and PZT, in order to enhance the piezoelectric performance of the drawn fibers^25–27^. Although a higher concentration of the piezoelectric fillers typically leads to improved piezoelectric properties, it would also cause flow instabilities that eventually result in fiber breakage. Thus, to enable the stable drawings, one should properly choose the concentration of the piezoelectric fillers.

This paper describes the material combinations and processing employed in the preparation of piezoelectric laminated generators and piezoelectric fibers. We also present a detailed study of the piezoelectric properties of the as-fabricated laminated generators and fibers. The laminated piezoelectric generators were assembled by sandwiching an electrospun piezoelectric mat between two conductive polymer films. The piezoelectric fibers were fabricated *via* drawing of the multilayer fiber preforms. In our choice of the materials for fiber fabrication we used two criteria. First, to obtain the fibers with high piezoelectric performance, the active material should have high piezoelectric coefficient. Second, to maintain the high degree of control over drawing of the kilometer-long piezoelectric fibers the materials in the fiber preform should be thermo-mechanically compatible. Thus, PVDF was chosen as the host material for the piezoelectric layers, as it is a low-cost, stable thermoplastic polymer that can exhibit relatively high value of the piezoelectric coefficient. BTO nanoparticles were impregnated into PVDF polymer to improve its piezoelectric properties. BTO-PVDF nanocomposite mats used in planar generators and fiber preform assembly were fabricated using electrospinning as this process allows fine-tuning of the thermo-mechanical properties of nanocomposites by changing the polymer molecular weight, the concertation of the BTO nanoparticles and the processing conditions. Carbon-impregnated low density polyethylene (C-LDPE) was selected as the conductive materials, as it is both thermoplastic and electrically conductive (volume resistivity: 2.2 *Ω*·*m*). The polycarbonate (PC) polymer was used as the fiber core, acting as the mechanical support for the active layers during fiber drawing. The resulting piezoelectric fibers typically feature ~10 bilayers of C-LDPE films and BTO-PVDF piezoelectric layers that are wrapped around a polycarbonate core (Fig. 1b,d). The two fiber electrode layers were extended to the opposite sides of the exposed fiber surface for convenience of the electrical connectorization (Fig. 1b,c). The piezoelectric fibers could effectively convert mechanical energy into electricity. Experimentally, a piezoelectric fiber (20 wt% BTO-PVDF; 10 cm length) could generate an open-circuit voltage of ~1 V and a short-circuit current of ~0.7 nA, when subjected to a 10 mm-bending displacement in a cyclic bend-release test (0.1 Hz). More importantly, the piezoelectric fiber retained its performance even after three days’ cyclic bend-release tests. Compared to other piezoelectric fibers^20–23, 28^, the fibers presented in this work feature a swiss roll structure of the piezoelectric layer, which considerably increases the active surface area and reduces the layer thickness, this resulting in considerably higher voltages and currents (and consequently electric powers) that can be generated by such fibers. Large-area piezoelectric textiles could be fabricated by incorporating the drawn fibers into woven fabrics, thanks to their excellent mechanical properties (as they are made only of plastics), and the use of low-cost, high volume fabrication techniques (fiber drawing). Finally, we conclude the paper by detailing several technology demonstrators with potential applications for powering personal electronics and wearable sensing in the smart garments, automotive and aerospace industries.

## Results

### Fabrication and characterization of the laminated piezoelectric generators

**Figure 2:**
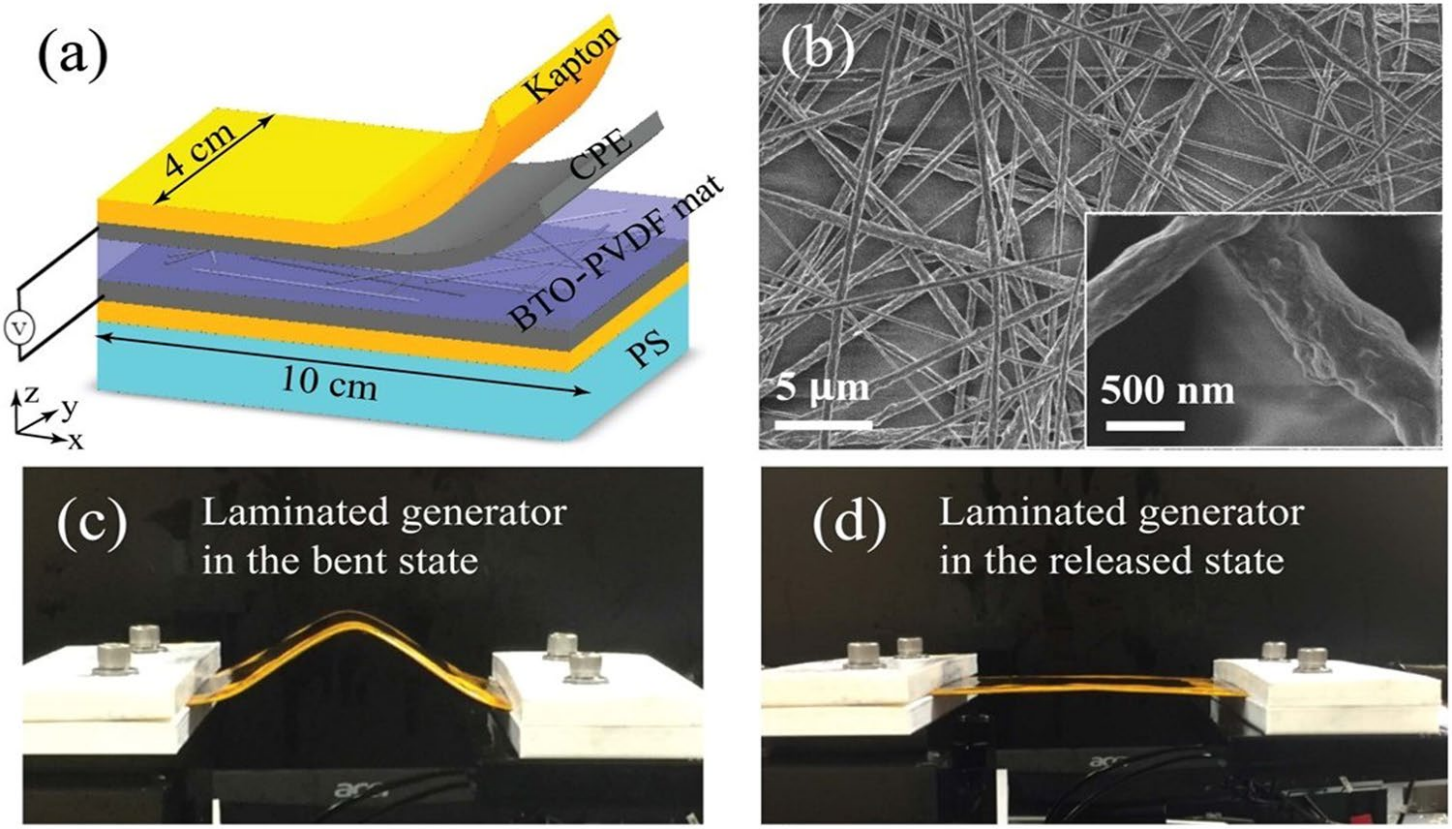
Design and performance of the laminated piezoelectric generators. (**a**) Schematic of a laminated BTO-PVDF generator that uses an electrospun piezoelectric mat. (**b**) SEM images of a BTO-PVDF mat at different magnifications. Insert: a magnified image of the BTO-PVDF nanocomposite. (**c**,**d**) Testing the laminated piezoelectric generator in its bent and released state.

Before we present fiber-based piezoelectric generators, we first detail piezoelectric properties of the BTO-PVDF nanocomposites used in fiber fabrication. BTO-PVDF mats (Fig. 2b) were fabricated *via* electrospinning with the BTO concentrations of 5, 10, 15 and 20 wt%. The FTIR^29–32^ and XRD^29, 31, 33^ studies of the BTO-PVDF mats are presented in Supplementary Note 1.

**Figure 3:**
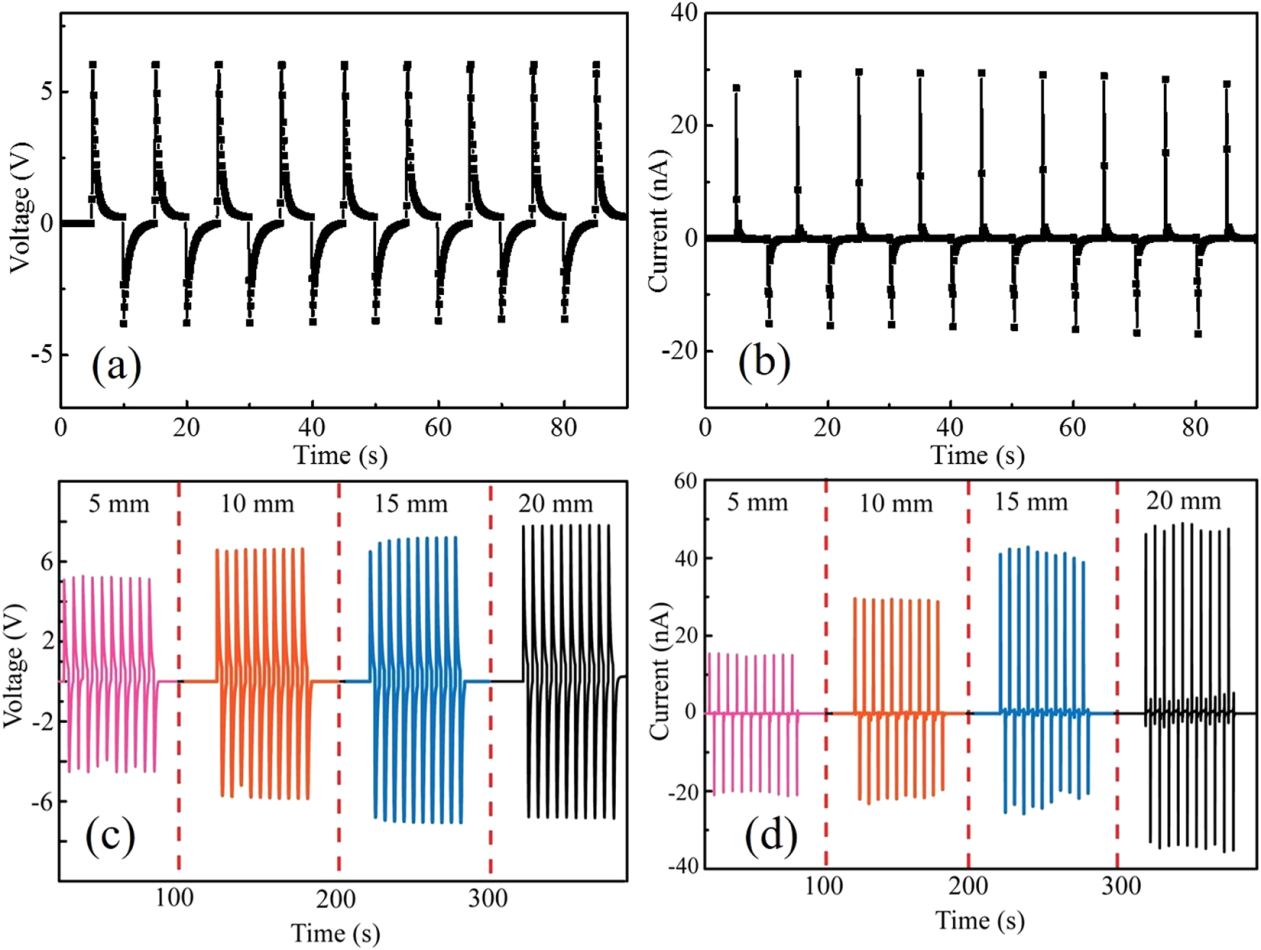
Performance of the laminated piezoelectric generators. (**a**) and (**b**) Show the generated output voltage of the laminated planar generator under bending cycle at different bending displacements (5, 10, 15, and 20 mm). (**c**) and (**d**) Show the output voltages and currents generated by a BTO-PVDF generator (20 wt.% BTO in the BTO-PVDF composite) when subjected to a 10 mm bending displacement.

A laminated piezoelectric generator was assembled by gluing an entire BTO-PVDF mat (thickness: 100 *μ*m) between the two C-LDPE electrode films (thickness: 75 *μ*m) using adhesive silver paste (Fig. 2a). And then the generators were poled in a silicone oil bath (80 °C) under the voltage of 5 kV for 24 hours. We then immobilized the laminated generator on a 1 mm thick polystyrene (PS) substrate using Kapton tape. Due to asymmetry in the generator structure, bending of the PS substrate would lead to the non-zero average strain in the BTO-PVDF mat. Experimentally, one end of the PS substrate was fixed, while the other end was horizontally displaced by a micropositioning stage, thus bending the generator. The laminated generators were cut into a rectangular shape with dimensions of 10 cm (in x direction) by 5 cm (in y direction), and the moving end of a generator is displaced along the x direction as shown in Fig. 2c,d. A typical voltage and current generated by a 20 wt% BTO-PVDF laminated generator under a 10 mm-bending displacement is shown in Fig. 3a,b. When the moving end of the generator was displaced by 5 to 20 mm, the corresponding open-circuit voltage increased from ~4 to ~8 V, and the short-circuit current increased from ~18 to ~50 nA (Fig. 3c,d).

The concentration of the BTO nanoparticles have a substantial effect on the piezoelectric properties of the mats. As the BTO concentration increased from 5 wt% to 25 wt%, the open-circuit voltage of the generator with its moving end displaced by 10 mm increased from ~0.5 V to ~8 V (Supplementary Fig. 1). We also note that the poling process that would align the dipoles is critical to actuate the piezoelectric function. Compared to the case of the poled generators, the unpoled ones generally have a piezoelectric voltage one order smaller (Supplementary Fig. 2). Finally, we tested the durability of the generator by continuously repeating the bend-release measurements for 3 days. The generator retained well its piezoelectric voltage and current throughout the whole test that comprised ~26000 bend/release cycles (Supplementary Fig. 3).

### Electro-mechanical model of the planar piezoelectric generator

**Figure 4:**
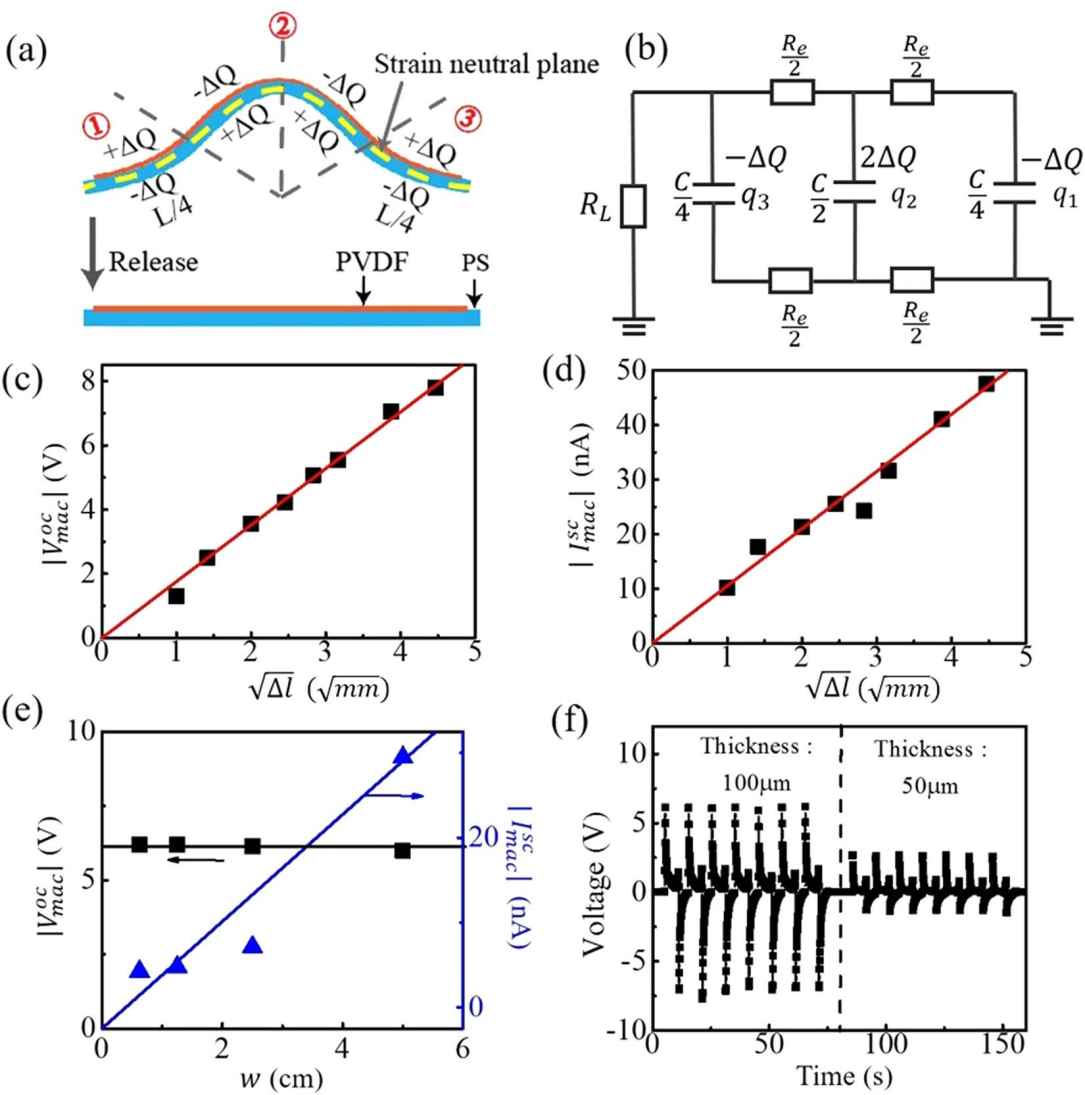
Electrical properties of the laminated piezoelectric generators under bending. (**a**) Schematic of charge separation in the piezoelectric mat under bending. (**b**) Equivalent electric circuit of the generator with a resistance load *R*_*L*_. (**c**,**d**) The measured output voltages (**c**) and currents (**d**) generated by a laminated generator (BTO concentration: 20 wt%) under different displacements ($\sqrt{{\rm{\Delta }}l}$) compared to the calculated ones (red lines) using Eq. (6). (**e**,**f**) The relationship between the output voltage (current) and piezoelectric mat width *w* (**e**), and piezoelectric mat thickness *d*_*p*_ (**f**).

In what follows we describe the fundamentals of operation of the laminated piezoelectric generators and study the relationships between the generated voltages and currents with the applied stage displacements, piezoelectric mat width and thickness. As an example, we focus on the electrical signals generated during the release part of the bending cycle. Thus, in the bent state, the generator can be divided into three regions with the lengths of *l*/4, *l*/2 and *l*/4, according to the sign of the curvature of the generator. In the first and the third regions the generator is under stress, while in the second region the generator is under strain (see Fig. 4a), therefore generating (Δ*Q*, −2Δ*Q*, Δ*Q*) fixed surface charges in the piezoelectric mat. In the equilibrium state of a generator, these charges are compensated by the opposite free charges in the electrodes. Therefore, at the beginning of the generator release (when the stress on the piezoelectric layer is suddenly released and the generator is flattened), mobile surface charges (−Δ*Q*, 2Δ*Q*, −Δ*Q*) will be present on the conductive electrodes. Consequent charge equilibration in the generator can then be approximated in terms of the effective electrical circuit shown in Fig. 4b, where *C* is the generator capacitance, *R*_*e*_ is a single electrode resistance, while *R*_*L*_ is the resistance of the load.

In the case of a planar piezoelectric mat of length *l*, width *w*, thickness *d*_*p*_, and dielectric constant ${\epsilon }_{p}$, the generator capacitance is $C=\frac{{\epsilon }_{p}{{\epsilon }}_{0}lw}{{d}_{p}}$. At the same time resistance of the electrode resistance that covers the mat is ${R}_{e}=\rho \frac{l}{w{d}_{e}}$, where *d*_*e*_ is the electrode thickness and *ρ* is the electrode material bulk resistivity. Using standard analysis of the basic electric circuits (Supplementary Note 2), we can derive the following expressions for the open circuit voltage ${V}^{oc}(t) \sim {V}_{max}^{oc}{e}^{-2\frac{t}{{\rm{\Delta }}{t}^{oc}}}$ and the short circuit currents ${I}^{sc}(t) \sim {I}_{max}^{sc}{e}^{-2\frac{t}{{\rm{\Delta }}{t}^{sc}}}$ with the following maximal values of the open circuit voltage and short circuit currents, as well as equilibration times Δ*t* ^*oc*^, and Δ*t* ^*sc*^:

1 $$\begin{array}{ll}\mathrm{Open}-\mathrm{circuit}\,\mathrm{voltage}: & {V}_{max}^{oc}={V}^{oc}(0)=-\frac{4{\rm{\Delta }}Q}{C}\\  & {\rm{\Delta }}{t}^{oc} \sim 2{\tau }_{0}\\ \mathrm{Short}-\mathrm{circuit}\,\mathrm{current}: & {I}_{max}^{sc}={I}^{sc}(0)=\frac{{\rm{\Delta }}Q}{{\tau }_{0}}\\  & {\rm{\Delta }}{t}^{sc} \sim 1.5279{\tau }_{0}\end{array}$$

where the time constant is defined as:

2 $${\tau }_{0}=\frac{{R}_{e}C}{8}=\frac{\rho {\epsilon }_{p}{{\epsilon }}_{0}}{8}\frac{{l}^{2}}{{d}_{p}{d}_{e}}$$

According to ref. 26, fixed charges Δ*Q* induced on the surface of a piezoelectric mat of length *l*/4 (see Fig. 4a) can be calculated as:

3 $${\rm{\Delta }}Q={d}_{31}{Y}_{p}\varepsilon w\cdot \frac{l}{4}$$

where *d*_31_ is the piezoelectric coefficient of a piezoelectric mat, *Y*_*p*_ is the Young’s modulus of the piezoelectric material, *w* is the width of the piezoelectric mat, and *ε* is the applied strain. From Eq. (1), we can then conclude that the peak open-circuit voltages have a liner relationship with the applied strain *ε*:

4 $$\begin{array}{c}|{V}_{max}^{oc}|=\varepsilon \frac{{d}_{31}{Y}_{p}wl}{C}=\varepsilon {d}_{p}\frac{{d}_{31}{Y}_{p}}{{\epsilon }_{p}{{\epsilon }}_{0}}\\ |{I}_{max}^{sc}|=\frac{{\rm{\Delta }}Q}{{\tau }_{0}}=2\varepsilon \frac{w\cdot {d}_{p}{d}_{e}}{l}\frac{{d}_{31}{Y}_{p}}{\rho {\epsilon }_{p}{{\epsilon }}_{0}}\end{array}$$

The evaluation of the applied strain *ε* is presented in the (Supplementary Note 3). By using a model for the buckling of thin beams, according to refs 15 and 34, the strain in the PVDF mat can be determined as:

5 $$\varepsilon \approx 2\pi \frac{{d}_{PS}\sqrt{{\rm{\Delta }}l}}{{l}^{3/2}}$$

where *d*_*PS*_ is the polymer substrate thickness, Δ*l* is the stage displacement, and *l* is the length of the laminated piezoelectric generator. Finally, remembering a relationship between the maximal open circuit voltage and the strain given by Eq. (4), we conclude that generated voltages and currents depend in a non-linear manner with the generator displacement, namely:

6 $$\begin{array}{c}|{V}_{max}^{oc}|=\varepsilon {d}_{p}\frac{{d}_{31}{Y}_{p}}{{\epsilon }_{p}{{\epsilon }}_{0}}=2\pi \frac{\sqrt{{\rm{\Delta }}l}{d}_{PS}{d}_{p}}{{l}^{3/2}}\frac{{d}_{31}{Y}_{p}}{{\epsilon }_{p}{{\epsilon }}_{0}}\\ |{I}_{max}^{sc}|=\frac{{\rm{\Delta }}Q}{{\tau }_{0}}=4\pi \frac{w\sqrt{{\rm{\Delta }}l}{d}_{PS}{d}_{p}{d}_{e}}{{l}^{5/2}}\frac{{d}_{31}{Y}_{p}}{\rho {\epsilon }_{p}{{\epsilon }}_{0}}\,\end{array}$$

To test our theoretical model against experimental measurements, we first characterized the laminated generators (*w*: ~5 cm; *l*: ~10 cm) under different stage displacements. In Fig. 4c,d we plot the peak voltages ($|{V}_{max}^{oc}|$) and currents ($|{I}_{max}^{sc}|$) as a function of the displacements ($\sqrt{{\rm{\Delta }}l}$) as calculated using Eq. (6) and observe an almost linear dependence in good agreement with theory. Note that, for each displacement, we repeated the bend-release cycles for 20 times; and the value of $|{V}_{max}^{oc}|$ and $|{I}_{max}^{sc}|$ are the average of these measured peaks. Then, we studied the relationship between the measured voltages (currents) and the piezoelectric mat width. We cut a generator along its longitudinal direction, and thus obtained smaller pieces with 1/2, 1/4 and 1/8 of the original widths and the same mat length. In Fig. 4e we find that the output voltage ($|{V}_{max}^{oc}|$) is independent of the piezoelectric mat width (*w*), while generated current increases with the mat width although the dependence is not linear and depends strongly on a sample. Finally, we investigated the relationship between the measured voltages and piezoelectric mat thickness. The thickness of the piezoelectric mat could be increased by using a longer electrospinning time, while keeping other processing conditions the same. Here, the electrospinning time and materials utilized in the fabrication of ~100 μm-thick piezoelectric mat was twice of that of ~50 μm-thick piezoelectric mat. In Fig. 4f we observe that the output voltage ($|{V}_{max}^{oc}|$) is indeed proportional to the thickness of piezoelectric mats (*d*_*p*_), which is again in accordance with our theory. Finally, from Eq. (6) and almost linear behavior of the output voltage to the displacements ($\sqrt{{\rm{\Delta }}l}$) value (see Fig. 4c) we can estimate the value of the *d*_31_ piezoelectric coefficient to be *d*_31_ ~ 13 pC/N which is lower than the *d*_31_ of BaTiO_3_ nanoparticles (~80 pC/N), but higher than that of the commercial piezoelectric PVDF films (~6 pC/N). Here, the dielectric constant (${\epsilon }_{p}$) of BTO/PVDF mat was estimated using the Bruggeman (BG) formulation^35^:

$$c\cdot \frac{{\epsilon }_{BTO}-{\epsilon }_{p}}{{\epsilon }_{BTO}+2{\epsilon }_{p}}+(1-c)\cdot \frac{{\epsilon }_{PVDF-}{\epsilon }_{p}}{{\epsilon }_{PVDF}+2{\epsilon }_{p}}=0\,$$

where ${\epsilon }_{p}$ is the complex effective dielectric constant of BTO suspension in the PVDF matrix, ${\epsilon }_{BTO}$ and ${\epsilon }_{PVDF}$ are the complex constants of the pure bulk BTO and PVDF, respectively. We note that thus found value of the *d*_31_ coefficient should only be considered as rough estimate as its value was found using the predictions of several approximate models designed to explain general trends in the electro-mechanical operation of the planar piezoelectric generators.

### Fabrication of the piezoelectric fibers *via* drawing of the multimaterial preforms

Figure 1 summarizes the fabrication process of the piezoelectric fibers. The first step is to assemble a fiber preform using the commercial and home-made materials. Two BTO-PVDF mats (thickness: 100 *μ*m) and C-LDPE films (thickness: 85 *μ*m) were co-rolled onto a hollow polycarbonate rod with an outer diameter of 2.54 cm, in order to create a multilayer cladding having ~10 alternating piezoelectric-conductive layers (Fig. 1b,c). After assembly, the structure was then vacuum consolidated into a solid preform at 110 °C. Subsequently, the resulting fiber preform was thermal-mechanically drawn into meters of piezoelectric microsturectured fibers using a fiber drawing tower. Here the BTO-PVDF mats were fabricated in-house *via* electrospinning. We differed the BTO concentrations in the BTO-PVDF mats from 5% to 20% with a 5 wt% interval, in order to investigate the effects of BTO nanoparticles on the properties of the final fibers. The C-LDPE films were purchased from Bystat International Inc. and had a volume resistivity of 2.2 *Ω*·*m* . Note that, the carbon fillers in the polyethylene matrix will be re-arranged, and thus the conductive network will be altered during fiber drawing. After drawing, the bulk resistivity of the polymer composite in the drawn fiber will be increased dramatically. Thus, it is important to optimize the drawing parameters. In the drawings of piezoelectric fibers, the drawing temperature is set at 190 °C and the drawing speed is 500 mm/min, at which conditions the formation of conductive network during fiber drawing could be facilited. Our measurements suggests that the volume resistivity of the conductive layers in the piezoelectric fiber is ~2.5 *Ω*·*m* . The application of the electrical field during drawing would also affect the piezoelectric properties of the drawn fibers, because the electrical poling together with mechanical stretching could promote the *β* phase transformation in the PVDF layer. In our experiments, a voltage of up to 5 kV was applied to the preform. However, the results suggested that the polarization during fiber drawing is not effective as the drawing time is too short. On the other hand, we find the application of the high voltage could effectively control the layer thickness in the drawn fibers, since the two conductive layers in the molten polymer were forced to pull together due to the strong electrical force. In this way, the piezoelectric fibers with several tens of nm layer thicknesses could be obtained. Optical images of fiber cross section (Fig. 1d) show that the multilayer structure retained well after drawing, while the layer thicknesses typically ranged from 5 to 20 *μ*m. The obtained fibers were then immersed in silicone oil bath (80 °C) for further polarization. After applying a voltage of 9 kV for 12 h, the fibers were slowly cooled down to room temperature.

### Characterization of the piezoelectric microstructured fibers

**Figure 5:**
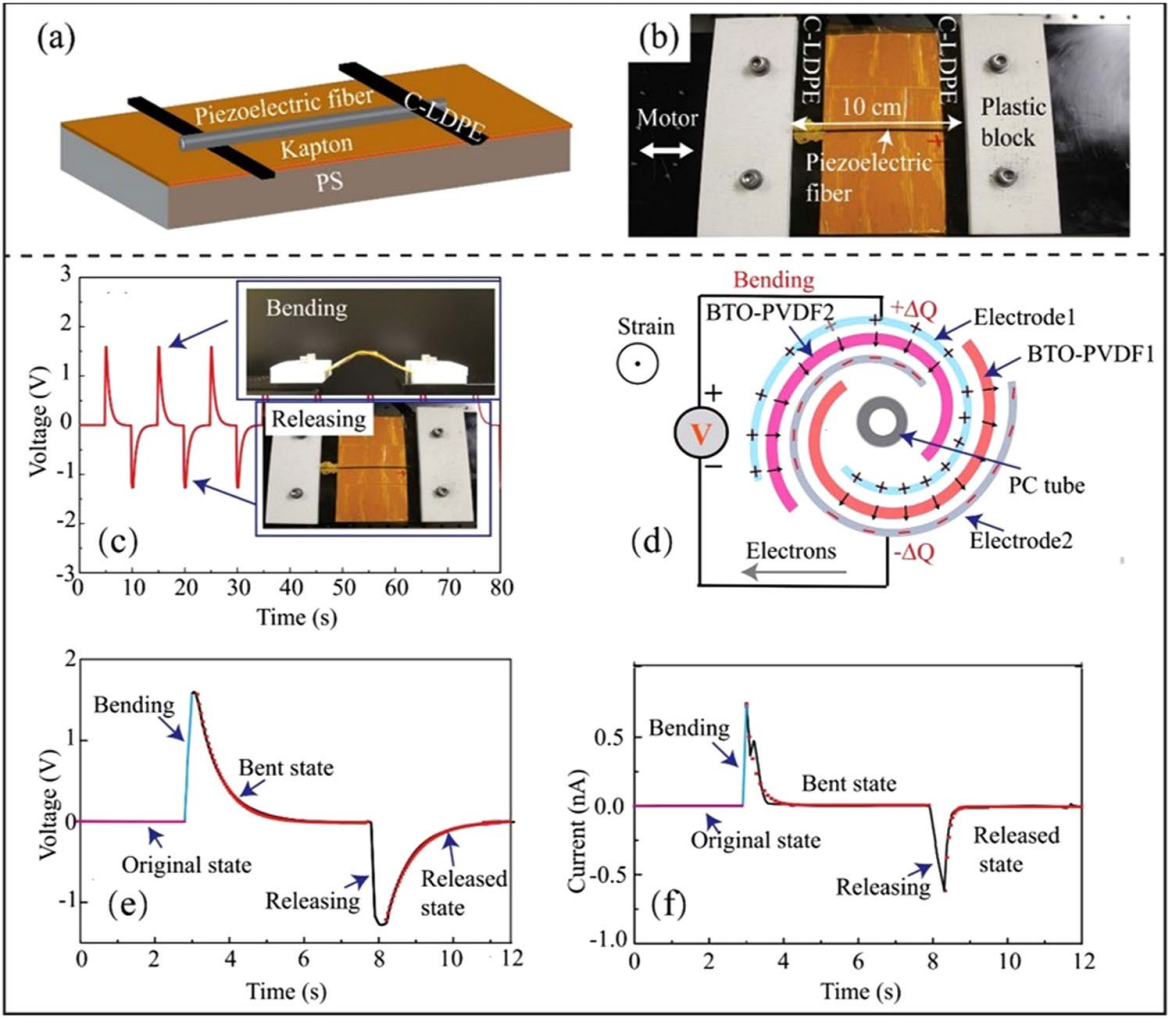
Design and working principles of the fiber-based piezoelectric generators. (**a**) Schematic of a BTO-PVDF fiber-based generator. (**b**) Setup for testing of microstructured fiber generator. (**c**) Measured voltage response of the piezoelectric fiber under cycling bending at 0.1 Hz. The top and bottom insets show photographs of the fiber during bending and release, respectively. (**d**) When mechanical strain is applied along the fiber by bending, the polarization density of the BTO-PVDF layer is changed and the electrons are forced to flow from one electrode to the other, thus generating voltage differential. (**e**,**f**) The open-circuit voltage (**e**) and the short-circuit current (**f**) of the piezoelectric fiber during the bend and release actions. Relaxation of the short circuit current and open circuit voltage on time can be described as single exponential decays with two distinct time constants (red curve fits).

We then characterized the performance of the piezoelectric microstructured fibers. A piezoelectric microstructured fiber (length: ~10 cm, diameter: ~1 mm) was used to assemble the fiber-based generator. As shown in Fig. 5a,b, two C-LDPE strips were used to connect the fiber electrodes. Particularly, one C-LDPE strip was glued to the top side of the fiber, while the other one was glued to the opposite side. Similar to the laminated piezoelectric generators, the fiber-based devices also consisted of a PS substrate (10 cm long, 1 mm thick) and Kapton tapes. Experimentally, the fiber-based generators were periodically bent and released in the horizontal direction using a linear motor. During the bend/release motions, the PS substrate worked as the bottom supporter and the Kapton tape covered the piezoelectric fibers. The mechanical strain was applied along the piezoelectric fiber by displacing one fiber end. The working principle of the fiber-based generators is discussed as follows. When the piezoelectric fiber is bent and released, positive and negative voltage spikes are observed (Fig. 5c). To explain this phenomenon, one needs to examine the charge separation mechanism and equivalent circuit model of the piezoelectric fiber. In the electrical poling, the dipoles of the piezoelectric domains in the BTO-PVDF layers are aligned in one direction. Due to the presence of the electric field of the dipoles, surface charges +Q and −Q are induced on the top and bottom electrode respectively. When a tensile stress is applied along the piezoelectric fiber, the polarization density of piezoelectric layers will change, thus inducing ±ΔQ changes in the surface charges of the fiber electrodes. In response to that, the electrons are forced to flow from one electrode to the other, thus generating voltage differential (Fig. 5d). In the bent state, the output voltage (current) gradually return to zero. Similarly, to the planar piezoelectric generators in the absence of the external load resistance, the charge relaxation processes in the generator can be considered as the RC charging/discharging with the two different time constants, one for the open-circuit voltage equilibration *τ*_*co*_ and the other for the short-circuit current equilibration *τ*_*sc*_.

**Figure 6:**
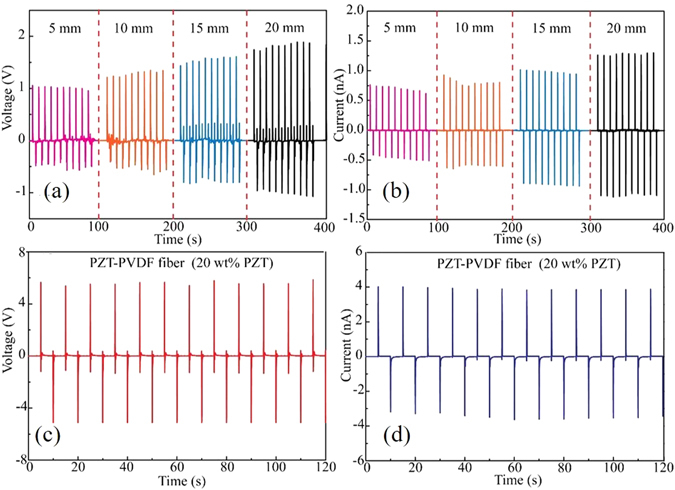
Performance of the piezoelectric microstructured fibers. (**a**) and (**b**) Show the generated output voltage of the fiber-based generator under bending cycle at different bending displacements (5, 10, 15, and 20 mm). (**c**) and (**d**) Show the output voltages and current sgenerated by a 10 cm-long PZT-PVDF fiber generator (20 wt.% PZT in the PZT-PVDF composite) when subjected to a 10 mm bending displacement.

The electrical outputs of the fiber-based generators are also affected by the BTO concentrations. Experimental results (Supplementary Fig. 4) suggest that the open-circuit voltage of the piezoelectric fiber-based generators increased form ~0.15 V to ~2.5 V when subjected to a 10 mm-bending displacement, while the BTO concentration in the piezoelectric fiber increased from 5 wt% to 25 wt%. However, we find that drawing fibers with BTO concentrations higher than 25 wt% is challenging. Within the fiber drawing process, the preform is softened above the glass transition temperature, and then pulled into a fiber at a specified speed. When the BTO concentration is too high, during drawing the nanoparticles will aggregate in the melt and form multiple macroscopic domains. While the preform diameter is being reduced, these domains serve as defect centers that cause flow instabilities that eventually result in fiber breakage. Thus, there is a trade-off between BTO concentration and the availability of fiber drawing process. All the drawings in this paper used the 20 wt% BTO concentration, since at this condition the drawing is stable while the piezoelectric functionality of the as-drawn fibers is maximized. Similar to the laminated generators, the performance of the fiber-based generators is also affected by the bending displacements. The piezoelectric voltage and current generated from the microstructured fibers increased from ~ 1 to ~1.7 V and ~ 0.7 to ~1.3 nA respectively, when the bending displacements increased from 5 mm to 20 mm (Fig. 6a,b). Besides, the electrical poling process is also required for the improvement of the piezoelectric fiber performance, since the poling can align the piezoelectric domains in the same direction. As stated in Supplementary Fig. 5, the output voltage of non-poled fibers was ~1.5 mV, which was much smaller than that of poled ones (~1 V). The mechanical stability of the piezoelectric fibers was estimated by conducting continually bend-release tests for 3 days. The amplitudes of output voltages and currents exhibit high stability for 25920 bend/release cycles. This phenomenon is probably attributed to the flexibility and robust of the polymer materials utilized in the fiber fabrication. The properties of the piezoelectric fibers could be improved by using piezoelectric materials with higher piezoelectric coefficients. According to the literature, PZT has a higher piezoelectric coefficient than BTO^36^. The PZT-PVDF (20 wt% of PZT in the PZT-PVDF composite layer) fibers were thus fabricated and then characterized using the same procedures as used for BTO-PVDF fibers. Experimentally, when subjected to the same bending displacements, the output signals generated from PZT composite fibers is ~4 times higher than that from BTO composite fibers (Fig. 6c,d). However, the toxicity of PZT may limit the potential applications.

### Examples of the potential applications of the piezoelectric fiber generators

**Figure 7:**
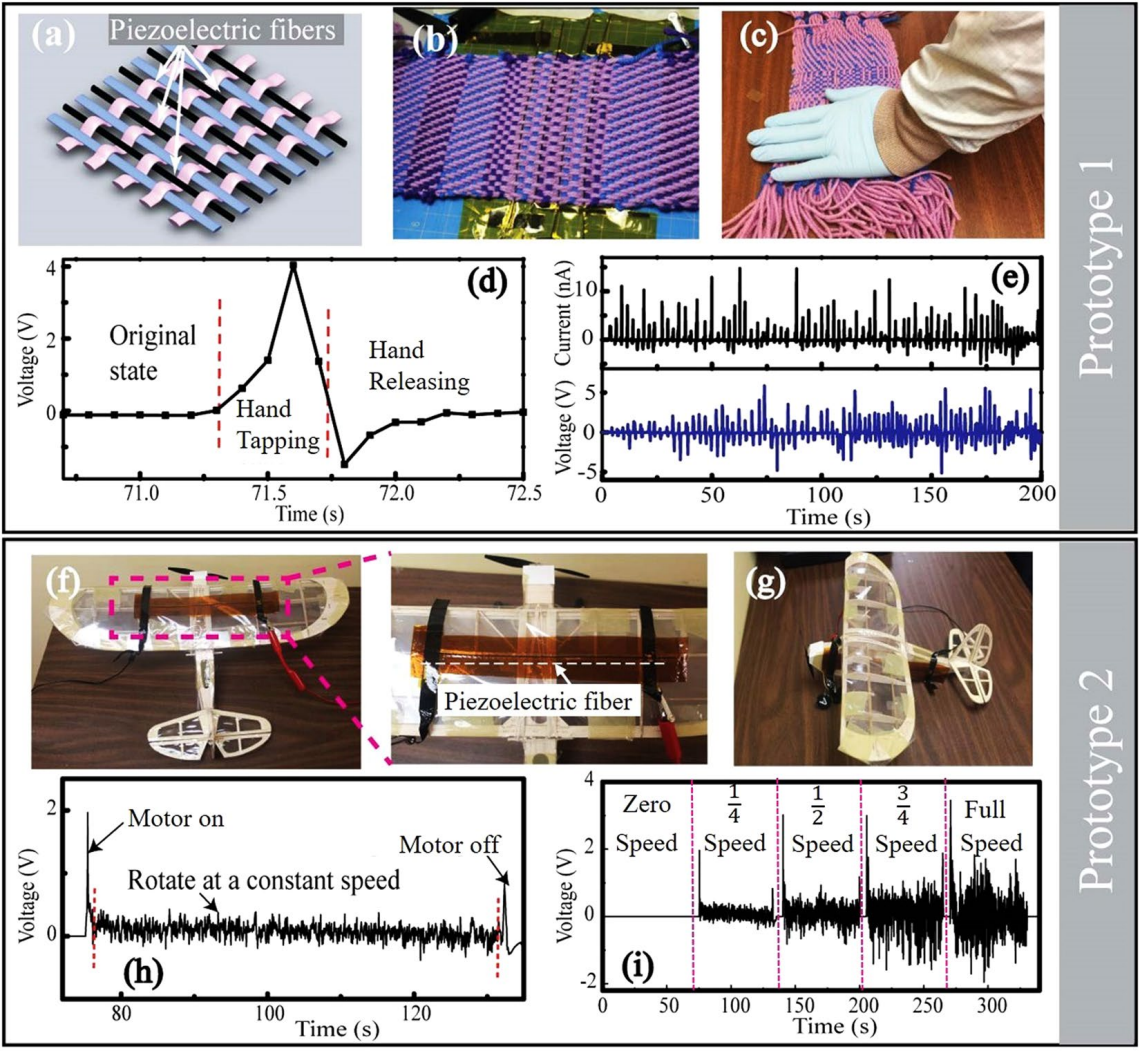
Potential applications of the piezoelectric fiber generators. (**a**,**b**) A cotton-based textile containing piezoelectric fibers woven using a Dobby loom. (**c**) Electrical properties of the piezoelectric textile actuated by the human hand tapping. (**d**) Open-circuit voltages of the piezoelectric textile in a hand tapping-releasing actions. (**e**) Open-circuit voltages and short-circuit currents generated by the piezoelectric textile during repeated hand tap-release motions. (**f**,**g**) Piezoelectric fibers implanted on the airplane wing (**f**) and the airplane body (**g**). (**h**) Open-circuit voltages generated by the piezoelectric fibers during rotation of the airplane propeller (**i**). (**h**) Open-circuit voltages generated by the vibrations induced by the airplane motor operation with the motor speeds set at zero, 1/4, 1/2, 3/4 of its maximum speed.

Piezoelectric microstructured fibers could be easily integrated into large-area cotton textiles using traditional textile fabrication techniques, thanks to the excellent mechanical properties of the drawn fibers. In this paper, the piezoelectric textiles were fabricated using a classic Dobby-loom (see Video 1). Here we discuss two prototypes of the fiber-based generators and demonstrate their possible applications as micro generators or sensors for the wearables and automotive/airspace applications. In the first prototype, four piezoelectric fibers (length: ~15 cm; diameter: ~1 mm) were woven into a cotton fabric and then connected in series (Fig. 7b). The piezoelectric textile could generate open-circuit voltages up to ~5 V and short-circuit currents of 2–10 nA, during the repeated irregular deformations caused by the human hand tap-release actions (Fig. 7d,e). In the second prototype, the piezoelectric fiber (length: ~15 cm; diameter: ~1 mm) was glued on the exterior of the airplane model. Note that, the tiny and flexible piezoelectric fibers could be implanted on the any parts of the airplane (Fig. 7f,g). During the tests, the airplane model was fixed on the wooden table. As we turned on the airplane motor, the rotation of the airplane propeller resulted in the irregular vibrations of the piezoelectric fibers, thus generating electric signal (see Video 2). The output voltages of the piezoelectric fibers are highly dependent on the rotation speed of the airplane motor. As the propeller rotation speed increased to the maximum, the open-circuit voltage of the piezoelectric fiber increased from 0 to 2 V (Fig. 7h,i).

## Conclusion

In this work, we have demonstrated both planar piezoelectric generators and piezoelectric fibers. The piezoelectric laminated generators were assembled first in order to study energy harvesting properties of the piezoelectric electrospun nanocomposites. Such planar generators can generate open-circuit voltages of up to 8 V and short-circuit voltage of up to 40 nA with an active area of several tens of cm^2^. Then, using electrospun nanocomposites as building materials, all-polymer piezoelectric microstructured fibers were fabricated using fiber drawing technique. The fabricated fibers feature a hollow PC core surrounded by a multilayer cladding consisting of the alternating BTO-PVDF and conductive C-LDPE layers. A swiss roll structure of the piezoelectric layer used in our fibers, considerably increases the active surface area and reduces the piezoelectric layer thickness, this resulting in high voltages of up to 6 V and currents of up to 4 nA that can be generated by our fibers with only several cm^2^ of the active area. Finally, we have demonstrated that the large-area piezoelectric textiles could be fabricated by incorporating piezoelectric fibers into the woven fabrics, thanks to the fiber excellent mechanical properties. The fabricated textiles and fibers were then used to highlight several of their potential applications as micro power generators or sensors in smart textile apparel and avionics.

## Methods

### Materials

The PVDF polymer used in this experiment was a semicrystalline PVDF (pellet, Sigma-Aldrich) which has a number average molecular weight of ~275,000. Dimethylformamide (DMF) and acetone solvent were purchased from Sigma-Aldrich. BTO nanoparticles (average diameter of 200 nm) were purchased from US Research Nanomaterials Inc. PZT micropowders (50–100 *μ*m, APC 850) were purchased from APC Inc.

### Preparation of polymer solutions

#### Preparation of BTO-PVDF suspensions

A good dispersion of inorganic particles in polymer matrix is necessary to achieve the best possible performance of the polymer nanocomposite^37^. We use the ultrasound to improve the dispersion of the BTO nanoparticles. A suspension of BTO-DMF was ultrasonically irradiated using a probe type sonicator (Fisher Scientific Inc.) at 100 W for 1 h. To prevent the DMF solvent from heating, the suspension was irradiated in 5 s intervals (3 s pulse on, 2 s pulse off). PVDF was swelled in acetone using a magnetic stirrer for 10 min. Then, the two suspensions were mixed together using a magnetic strirrer while being heated at 100 °C for 1 h. Finally, the mixed suspension was ultrasonically irradiated at at 75 W in 5 s intervals (3 s pulse on, 2 s pulse off) for 15 min, and then transfered to a vacuum chamber to remove the air bubbles. Here, the PVDF concentration was 20%, the BTO concentrations in the BTO-PVDF nanocomposites were ranges from 5 to 25 wt% with a 5 wt% variation. The DMF/acetone volume ratio was 2/3.

#### Preparation of PZT-PVDF Suspensions

The PZT micropowders were milled using a ball-milling machine (MSK-SFM-2, MTI Corporation). The milling time is 10 h, while the milling speed is 200 rpm. The weight ratio of ball-to-PZT was 20:1. The as-milled PZT micropowders were then utilized in the preparation of PZT-PVDF suspensions, following the same procedures proposed for the preparation of BTO-PVDF suspensions.

### Electrospinning

The Electrospinning Workstation (MSK-NFES-3, MTI Corporation) consists of a high voltage supply, a glass syringe (20 mL) with a blunt metallic needle (22 gauge) and a grounded metallic drum (diameter: 5 cm). The drum rotating at a speed of 200 rpm was used to collect the piezoelectric nanofibers. In the electrospinning, the polymer suspensions were charged by a high voltage of 15 kV, and the distance between the needle tip and the drum collector is 15 cm. Electrospinning was done with an ejection rate of 1 *ml*/*h* from the syringe. The temperature in the electrospinning chamber was controled at 25 °C. After the electrospining, the electrospun mats were vacuum dried at room temperature for 24 h.

### Preform and fiber fabrication

The preform and fiber structure is illustrated in Fig. 1b,c. The fabrication of the preform started with co-rolling of four alternating BTO-PVDF electrospun mats and C-LDPE conductive films. The preform was then vacuum consolidated at a temperature of 110 °C. The as-assembled preform was subsequently thermally drawn in a two temperature-zone verticle furnace. The bottom temperature is 190 °C, while the top temperature is 150 °C. The drawing speed was set at 500 mm/min. And an air-pressure of 3 mbar is used to maintain the fiber core during the drawing.

## Electronic supplementary material

Piezoelectric Microstructured Fibers via Drawing of Multimaterial Preforms

Video 1-the fabrication of piezoelectric textile

Video 2-piezoelectric fiber implanted on the airplane model
