# Supplementary material for: Piezoelectric Microstructured Fibers via Drawing of Multimaterial Preforms
Source: Sci Rep. 2017 Jun 6;7:2907. doi: 10.1038/s41598-017-01738-9 (PMC5460216; doi:10.1038/s41598-017-01738-9)
Supplement: Supplementary file 1 — Piezoelectric Microstructured Fibers via Drawing of Multimaterial Preforms [file 41598_2017_1738_MOESM1_ESM.pdf]

Supplementary Figures and Notes

Piezoelectric Microstructured Fibers *via* Drawing of Multimaterial Preforms

Xin Lu, Hang Qu and Maksim Skorobogatiy

École Polytechnique de Montréal, Montreal, Québec, H3T 1J4, Canada
Correspondence to Maksim Skorobogatiy.
E-mail: maksim.skorobogatiy@polymtl.ca

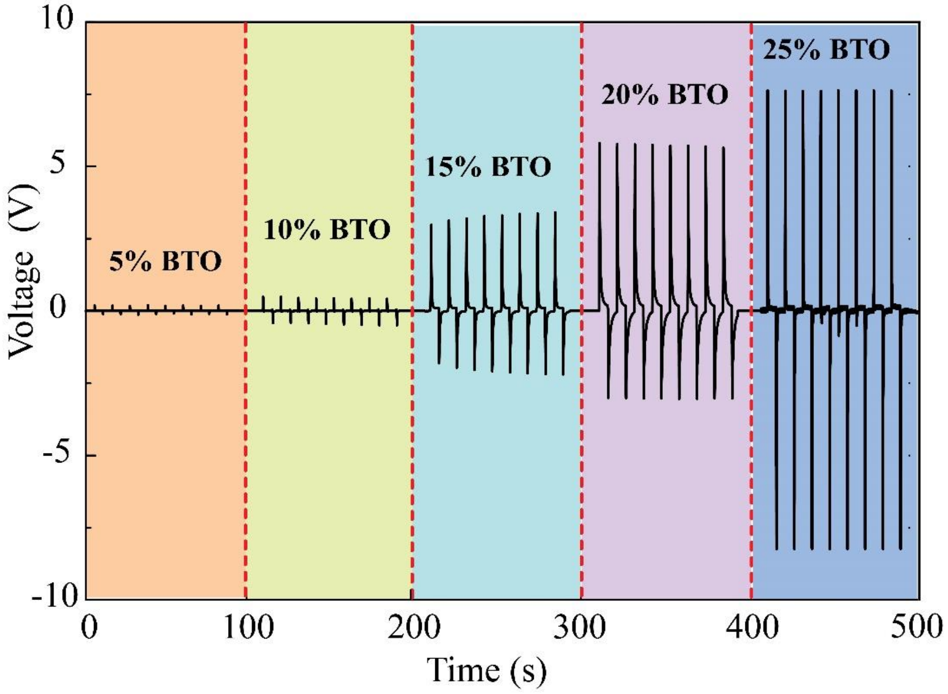

Supplementary Figure 1| Open-circuit voltage generated by the laminated BTO-PVDF generators with the BTO concentration of 5, 10, 15, 20, and 25 wt%. 10 mm-displacement was used in all tests.

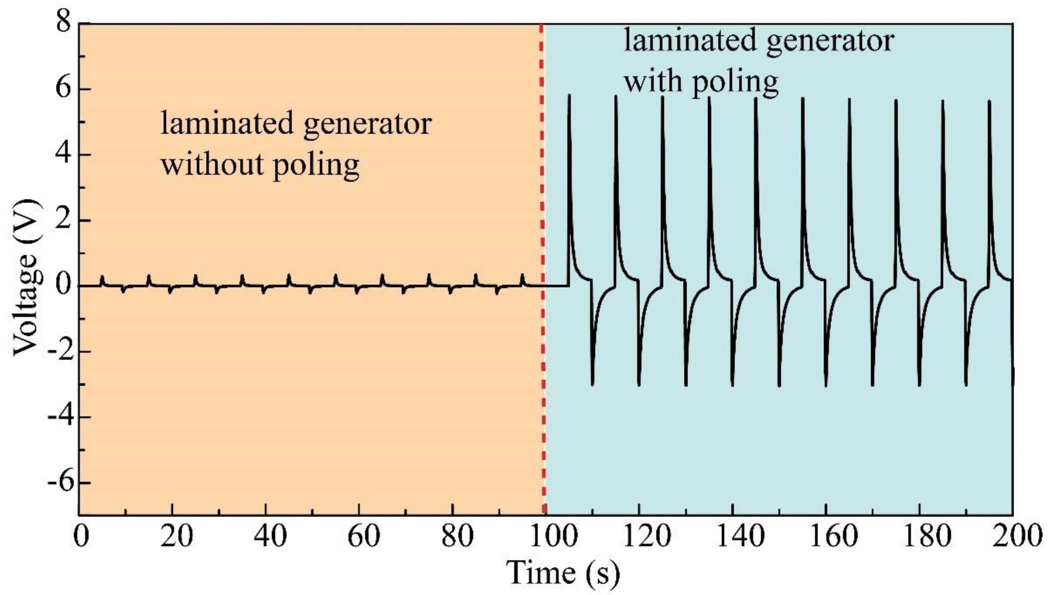

Supplementary Figure 2| Comparison of the open-circuit voltage generated by the poled laminated BTO-PVDF (20 wt% BTO) generator to that of the unpoled one.

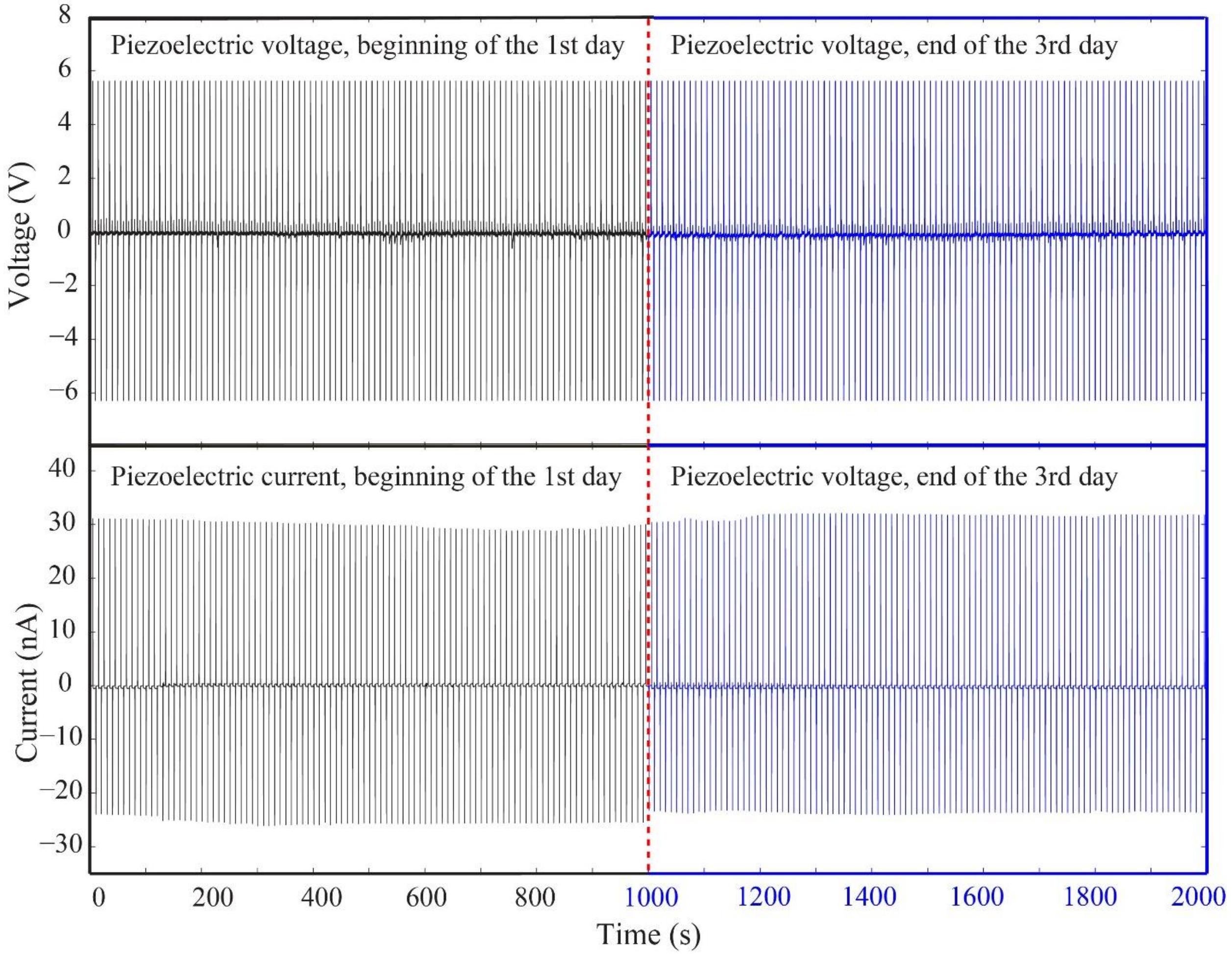

Supplementary Figure 3| A durability test was carried out for the laminated BTO-PVDF generator (20 wt% BTO) by continuously repeating the bend-release test for 3 days. In each bend-release motion, the moving end of the generator was displaced by 10 mm. The open-circuit voltage and short-circuit current generated in a 1000 s period at the beginning of in the first day and at the end of the third day are shown. Overall, 25920 bend/release cycles were performed during 3 days.

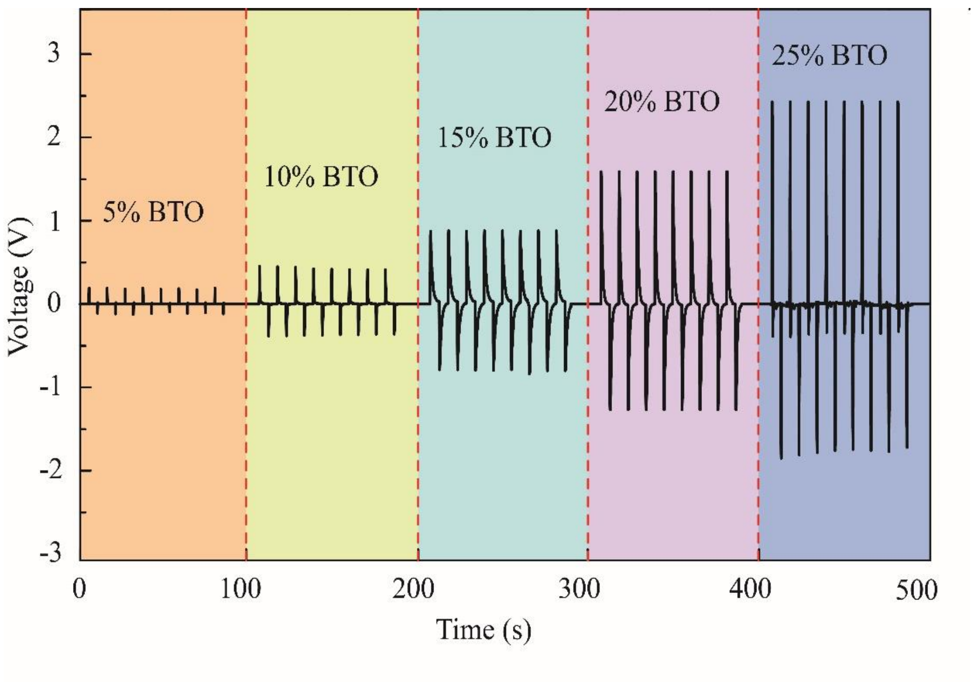

Supplementary Figure 4| Open-circuit voltage generated by BTO-PVDF microstructured fiber with the BTO concentration of 5, 10, 15, 20, and 25 wt%.

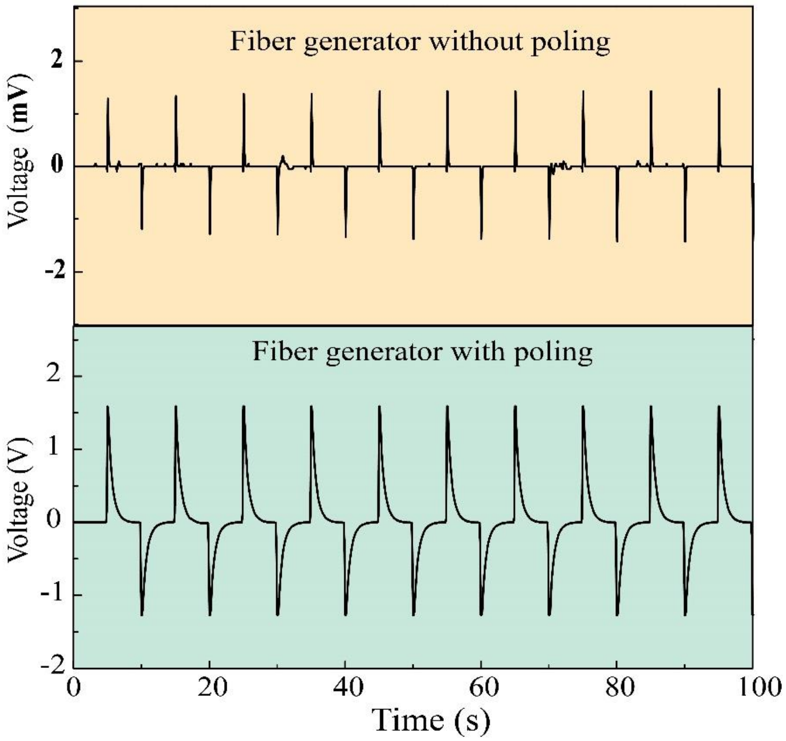

Supplementary Figure 5| Comparison of the open-circuit voltages generated by the poled BTO-PVDF microstructured fiber and the unpoled one.

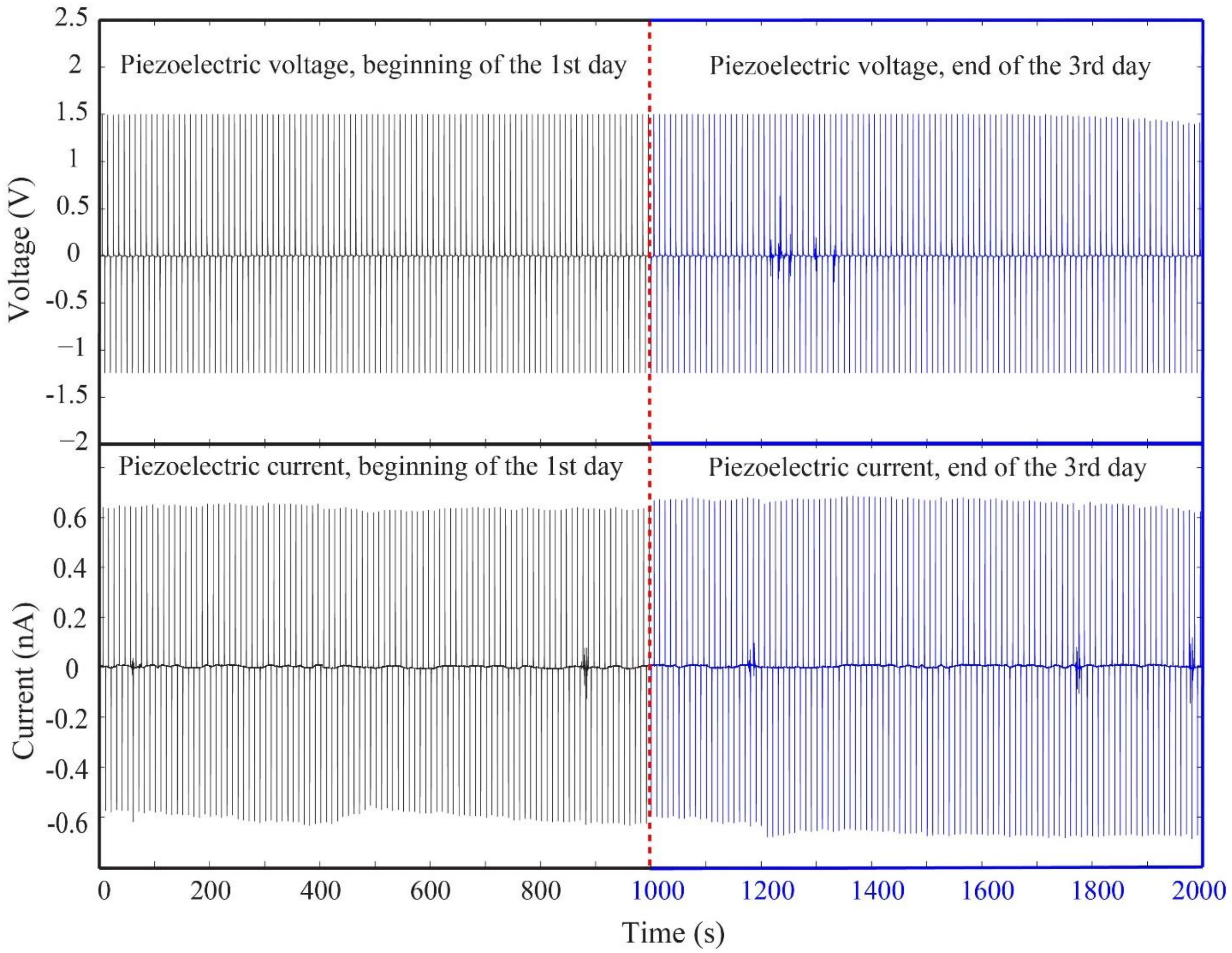

Supplementary Figure 6| A durability test was carried out for the BTO-PVDF microstructured fiber (20 wt% BTO in BTO-PVDF composite) by continuously repeating the bend-release test for 3 days. The open-circuit voltage and short-circuit current generated in a 1000 s period at the beginning of the first day and at the end of the third day are shown.

Supplementary Note 1. Piezoelectric properties of the BTO-PVDF mats

In the following we detail dependence of the piezoelectric properties of the BTO-PVDF mats on the concentration of the piezoelectric nanofillers using FTIR and XRD techniques.

FTIR of BTO-PVDF mats

Fourier transform infrared (FTIR) spectra were recorded on a FTIR spectrometer (FTLA2000-104, ABB Inc.). The vibration bands at 761 (CF_2 bending and skeletal bending), 796 (CH_2 rocking), 870, 974, 1146 and 1383 cm^{-1} correspond to α phase; whereas vibration bands at 840 and 1274 cm^{-1} (CH_2 rocking) are assigned to β phase[29-32].

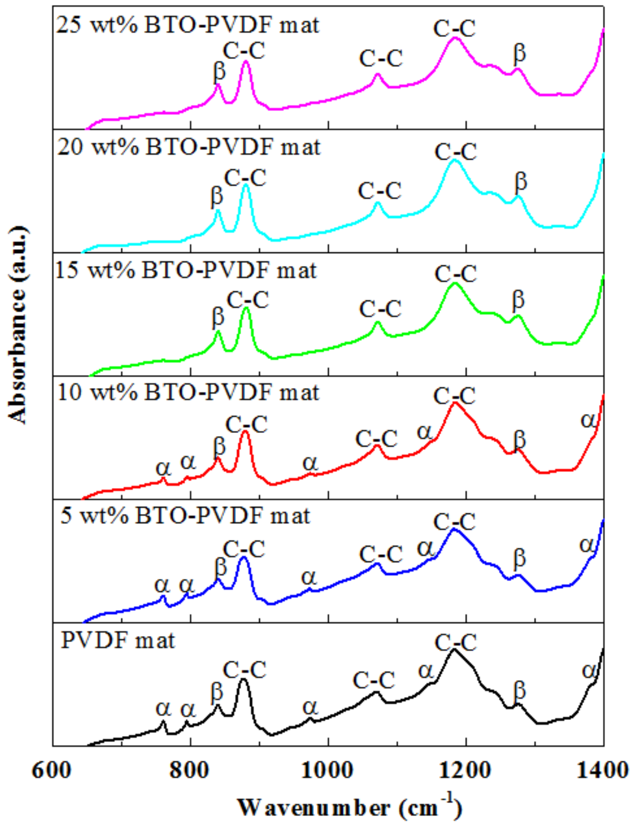

Supplementary Figure 7| FTIR spectra of BTO-PVDF electrospun mats at different BTO concentrations.

As shown in Supplementary Fig. 7, when the BTO concentration is higher than 15%, α phase could be hardly observed. When the BTO concentration is lower than 10%, α phase and β phase coexist in the nanocomposite. The β phase content, F(β), in the PVDF mats was calculated using the following Eq.:[29]

$$F_{(\beta)} = \frac{X_\beta}{X_\alpha + X_\beta} = \frac{A_\beta}{\frac{K_\beta}{K_\alpha} A_\alpha + A_\beta}$$

Where A_α and A_β are the absorbance at 761 cm^{-1} and 840 cm^{-1} respectively, and X is the degree of crystallinity of each phase. K_α and K_β are the absorption coefficient at the respective wavenumber, which are 6.1×10^4 and 7.7×10^4 cm^2 mol^{-1}. From calculation, we find that the β crystal phase content increased from 56% to 70%, when the BTO concentration increases from 0 to 10 wt%.

XRD of BTO-PVDF mats

The X-ray diffraction patterns were recorded by a Bruker D8/Discover diffractometer equipped with a standard sealed tube producing Cu radiation (λ = 1.54178 Å) running at 40 kV and 40 mA. The peaks at 2θ values of 18.5°, 20.4° are indexed to the α(020), β(200/110) reflections of PVDF respectively[29,31], while the other characteristic peaks can be assigned to the tetragonal phase of $BaTiO_3$[33].

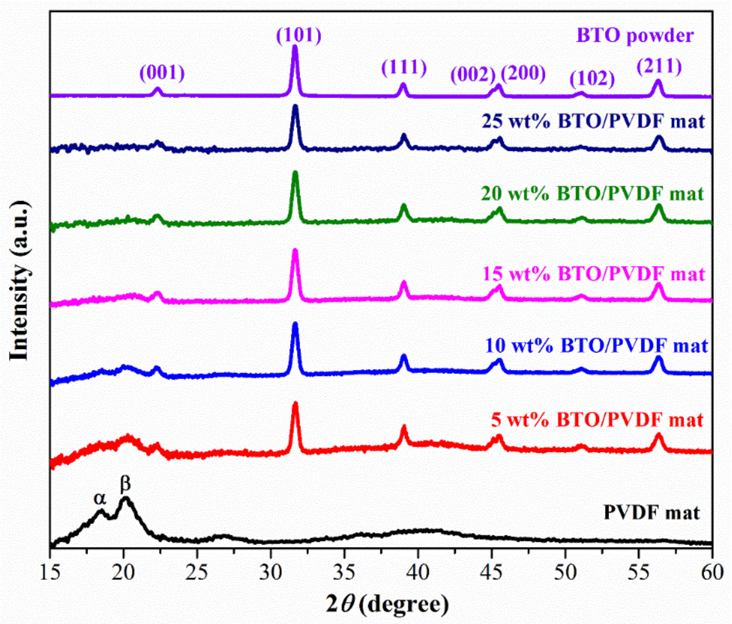

Supplementary Figure 8| The XRD patterns of the PVDF mats featuring different BTO concentrations

For the nanocomposites, when the BTO concentration is lower than 15%, we can observe a weak peak at 20.4° (characteristic of the β phase in PVDF). However, when the BTO concentration is higher than 15%, the two peaks that are characteristic to scattering in amorphous PVDF (see the left hand side of the bottom panel in Supplementary Fig. 8) cannot be clearly observed, which is ascribed to the shielding effect due to high intensity diffraction peaks in BTO nanocrystals.

Supplementary Note 2. Effective electric circuit of the planar piezoelectric generator

Analysis of the effective electric circuit presented in Fig. 4b, lead to the following differential equations that govern time dynamics of the individual capacitor discharges:

$$\frac{q_3}{C/4}+\frac{R_e}{2}\cdot \dot{q}_3+\frac{R_e}{2}\cdot(\dot{q}_2+\dot{q}_3)+R_L(\dot{q}_1+\dot{q}_2+\dot{q}_3)=0$$

$$\frac{R_e}{2}(\dot{q}_1+\dot{q}_2)+\frac{R_e}{2}\dot{q}_1+\frac{q_1}{C/4}+R_L(\dot{q}_1+\dot{q}_2+\dot{q}_3)=0$$

$$\frac{R_e}{2}(\dot{q}_1+\dot{q}_2)+\frac{q_2}{C/2}+\frac{R_e}{2}\cdot(\dot{q}_2+\dot{q}_3)+R_L(\dot{q}_1+\dot{q}_2+\dot{q}_3)=0 \qquad (1)$$

Solution of which has to also satisfy the initial conditions:

$$(q_1,q_2,q_3)_{t=0}=(-\Delta Q,2\Delta Q,-\Delta Q) \qquad (2)$$

Assuming solution of the system of linear differential equations (1) in the form $\bar{q}=\bar{q}_\lambda e^{-\lambda t}$ (λ is a constant), one can find particularly simple solutions that satisfy both (1) and initial conditions (2) in the case of an open circuit $R_L=\infty$, and short circuit $R_L=0$, which results in the following expressions for the open circuit voltage $V^{oc}(t)$ and the short circuit currents $I^{sc}(t)$:

Open-circuit voltage: $V^{oc}(t)=-\frac{4\Delta Q}{C}e^{-\frac{t}{\tau_0}}$

$$V^{oc}_{max}=V^{oc}(0)=-\frac{4\Delta Q}{C}$$

$$\Delta t^{oc}\sim 2\tau_0$$

Short-circuit current: $I^{sc}(t)=\frac{\Delta Q}{\tau_0}(1.1707e^{-\frac{1.3090}{\tau_0}t}-0.1707e^{-\frac{0.1910}{\tau_0}t})$

$$I^{sc}_{max}=I^{sc}(0)=\frac{\Delta Q}{\tau_0}$$

$$\Delta t^{sc}\sim 1.5279\tau_0 \qquad (3)$$

Where the time constant is defined as:

$$\tau_0 = \frac{R_e C}{8} = \frac{\rho \epsilon_p \epsilon_0}{8} \frac{l^2}{d_p d_e} \tag{4}$$

From Eq. (3) one can then calculate the effective generator resistance $V_{max}^{oc}/I_{max}^{sc} = \frac{R_e}{2}$.

Note 3. Mechanical analysis of the laminated generators under bending

Here we discuss in more details evaluation of strain ε in the bent laminated generators. Consider, for example, the second region in Fig. 4a of length $\frac{l}{2}$ and suppose that it has a fixed curvature $1/r$. Then, the strain can be calculated using a theoretical model[15]. In that model (see Supplementary Fig. 9), the strain neutral plain can be calculated by the Eq. (5):

$$Y_1 d_1 y_1 + Y_2 d_2 y_2 + Y_3 d_3 y_3 + Y_4 d_4 y_4 = 0 \qquad (5)$$

Where Y's and d's are the Young's modulus and the thicknesses of various films in the generator structure, while the distances between the centers of the four layers (Kapton, PVDF mat, Kapton and PS substrate) and the neutral plane are y_1, y_2, y_3 and y_4 (counted from top down). Assuming that the substrate (PS) thickness dominates $d_4 \gg d_{1,2,3}$, while Young's modulus of the materials are comparable to each other then $y_2 \sim d_{PS}/2$.

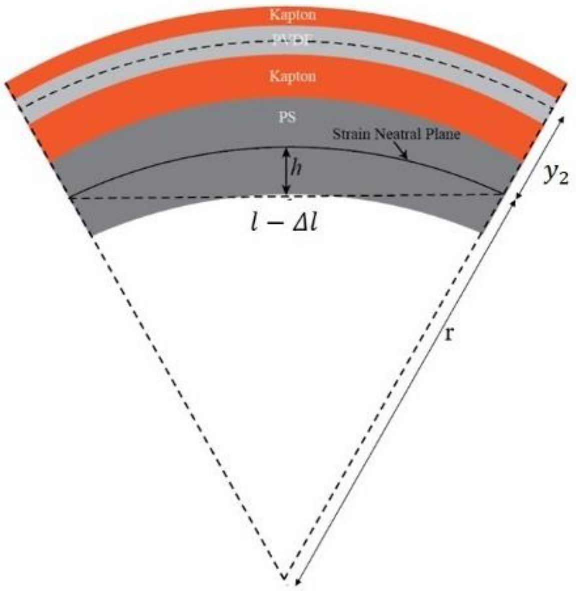

Supplementary Figure 9| Strain calculation in the laminated piezoelectric generators under bending.

Using a model for the buckling of thin beams (see Supplementary Fig. 10), according to ref. 34 the beam generator shape and the corresponding curvature (at the point of maximum deflection) for relatively large beam displacements ($l \gg \Delta l \gg d_{PS}^2/l$) are given by:

$$h(x) = \frac{A}{2}\left(1 + cos\left(\frac{2\pi x}{l-\Delta l}\right)\right);\ A \approx \frac{2}{\pi}\sqrt{\Delta l \cdot l}$$

$$\frac{1}{r} = \left.\frac{h''}{(1+(h')^2)^{3/2}}\right|_{x=\frac{l-\Delta l}{2}} \approx 2\pi^2\frac{A}{l^2} \approx 4\pi\frac{\sqrt{\Delta l}}{l^{3/2}} \qquad (6)$$

While the strain in the PVDF mat can be determined from ref.15 as:

$$\varepsilon = \frac{y_2}{r} \approx 2\pi\frac{d_{PS}\sqrt{\Delta l}}{l^{3/2}} \qquad (7)$$

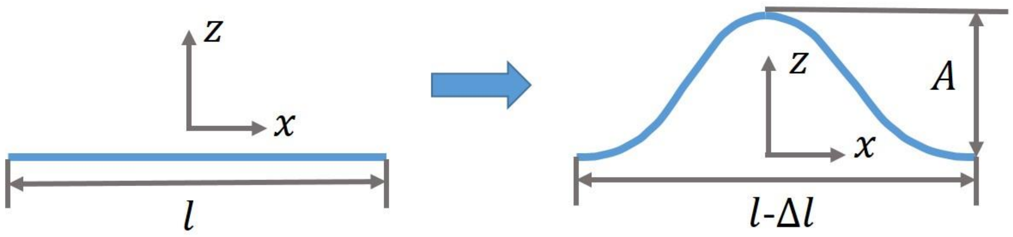

Supplementary Figure 10| Schematic diagram of mechanics model for the planar piezoelectric generators under bending.

Reference

15 Lee, M. *et al.* A hybrid piezoelectric structure for wearable nanogenerators. *Advanced Materials* **24**, 1759-1764, doi:10.1002/adma.201200150 (2012).

29 Shao, H., Fang, J., Wang, H. & Lin, T. Effect of electrospinning parameters and polymer concentrations on mechanical-to-electrical energy conversion of randomly-oriented electrospun poly(vinylidene fluoride) nanofiber mats. *RSC Advances* **5**, 14345-14350, doi:10.1039/C4RA16360E (2015).

30 Yu, S. *et al.* Formation Mechanism of β-Phase in PVDF/CNT Composite Prepared by the Sonication Method. *Macromolecules* **42**, 8870-8874, doi:10.1021/ma901765j (2009).

31 Sita, M. D., Siliang, W., Michael, J. & Treena Livingston, A. Structural changes in PVDF fibers due to electrospinning and its effect on biological function. *Biomedical Materials* **8**, 045007 (2013).
32 Karan, S. K., Mandal, D. & Khatua, B. B. Self-powered flexible Fe-doped RGO/PVDF nanocomposite: an excellent material for a piezoelectric energy harvester. *Nanoscale* **7**, 10655-10666, doi:10.1039/C5NR02067K (2015).
33 Lee, H.-W., Moon, S., Choi, C.-H. & Kim, D. K. Synthesis and size control of tetragonal barium titanate nanopowders by facile solvothermal method. *Journal of the American Ceramic Society* **95**, 2429-2434, doi:10.1111/j.1551-2916.2012.05085.x (2012).
34 Song, J. *et al.* Mechanics of noncoplanar mesh design for stretchable electronic circuits. *Journal of Applied Physics* **105**, 123516, doi:10.1063/1.3148245 (2009).
